# The Use of the Nematode *Caenorhabditis* *elegans* to Evaluate the Adverse Effects of Epoxiconazole Exposure on Spermatogenesis

**DOI:** 10.3390/ijerph13100993

**Published:** 2016-10-08

**Authors:** Yunhui Li, Minhui Zhang, Shaojun Li, Rongrong Lv, Pan Chen, Ran Liu, Geyu Liang, Lihong Yin

**Affiliations:** 1 Key Laboratory of Environmental Medicine Engineering Ministry of Education, School of Public Health, Southeast University, Nanjing 210009, Jiangsu, China; mhzhang@seu.edu.cn (M.Z.); rronglv@163.com (R.L.); ranliu@seu.edu.cn (R.L.); lianggeyu@163.com (G.L.); lhyin@seu.edu.cn (L.Y.); 2 Department of Molecular Pharmacology, Albert Einstein College of Medicine, Bronx, New York, NY 10461, USA; Shaojun.li@einstein.yu.edu (S.L.); Pan.Chen@einstein.yu.edu (P.C.)

**Keywords:** epoxiconazole, spermatogenesis, TGF-β, *Caenorhabditis elegans*

## Abstract

There is increasing evidence that epoxiconazole exposure can affect reproductive function, but few studies have investigated adverse effects on spermatogenesis. The nematode *Caenorhabditis elegans* (*C. elegans*) was used in our study to assess effects of epoxiconazole on spermatogenesis in male nematodes after 48 h of exposure to concentrations of 0.1, 1.0, or 10.0 μg/L. The results demonstrated that epoxiconazole exposure affected spermatogenesis, decreasing the number of total germ cells, mitotic cells, meiotic cells and spermatids, spermatid diameter, and cross-sectional area, and inducing mitotic germ cell proliferation arrest, premature entry into meiosis, and sperm activation inhibition; however, sperm transfer showed no abnormal changes. In addition, the results showed that epoxiconazole activated the transforming growth factor-β (TGFβ) signaling pathway and increased the expression levels of gene *daf-1*, *daf-3*, *daf-4*, *daf-5* and *daf-7* in nematodes. We therefore propose that epoxiconazole acts by activating the TGFβ signaling pathway, leading to the impairment of spermatogenesis and the consequent decline in male fertility.

## 1. Introduction

Triazole fungicides are the most efficient class of pesticides and have been in rapid development since the 1970s, receiving worldwide recognition. Epoxiconazole (CAS-No. 133855-98-8) is a commonly used member of the triazole class of plant protection products, and its environmental residue in water has been detected at concentrations up to 7.7 μg/L [1]. Previous studies have suggested that epoxiconazole is associated with renal diabetes, cardiovascular disease [2,3], and an increased risk of development toxicity [4,5]. In addition, epoxiconazole exposure alters reproductive parameters including copulation, fertilization, and gestational length and causes androgens disorders [2,6], demonstrating that epoxiconazole acts as a male reproductive toxicant [7,8]. Epoxiconazole may damage the seminiferous tubule causing a decrease in the number of spermatocytes, spermatogonia, and spermatids [9]. Further, it is suggested that epoxiconazole can affect the activity of the genes sterol 14α-demethylase (encoded by the CYP51 gene) and aromatase (encoded by the CYP19 gene) [10,11], which are required for membrane fluidity and integrity of fungal cells, and these genes also modulate the expression and function of mammalian cytochrome P450 enzymes and are highly expressed in germ cells, playing an important role in spermatogenesis [12,13]. However, little information is available concerning the potential toxicity of epoxiconazole on male spermatogenesis.

Spermatogenesis is a complex, highly organized, and regulated process that creates sperm from initially undifferentiated germ cells, and involves mitosis, meiotic entry, meiosis, spermiogenesis, and motility [14]. Errors in any process during spermatogenesis can result in failure of fertilization, which significantly contributes to infertility and miscarriages in humans. The process is regulated by a variety of signaling pathways, and the transforming growth factor β (TGF-β) pathway is one of the most critical [15,16,17]. Thus, we hypothesized that epoxiconazole disrupts the process of spermatogenesis via the TGFβ pathway. The period of time required for germ cells to complete this process including proliferation, differentiation, gamete formation, and fertilization is quite long, making it impossible to monitor the entire process of spermatogenesis in mammalian models [18]. According to a study by Susiarjo [19], the processes of spermatogenesis and sperm maturation are difficult to reproduce in cell culture systems, which have poor integrity and lack continuity. Therefore, it is particularly important to find suitable animal models for the study of spermatogenesis.

*Caenorhabditis elegans*, which is endowed with a highly differentiated yet simple reproductive system, is an amenable system to study spermatogenesis [20]. In particular, the transparency of the body allows direct observation of the processes of germ cell formation including proliferation, meiosis, and the formation of gametes via fluorescence microscopy. Importantly, *C. elegans* shares an evident degree of gene conservation with humans in the areas of spermatogenesis and signaling pathways such as TGFβ, making our experiments more convincing [21,22]. Of course, the accuracy of the extrapolation of nematode experimental results in mammals is uncertain. Nevertheless, *C. elegans* has certain advantages in specific mechanism research and preliminary screening tests; consequently, it can be a useful complement to the use of mammalian models and cell culture experiments.

Here, we present the results of a series of experiments to test the hypothesis that exposure to epoxiconazole results in reproductive toxicity and that this is due to impairment of spermatogenesis. Further, we explored the relationship between the TGFβ signaling pathway and the impairment of spermatogenesis induced by epoxiconazole. To our knowledge, the present study is the first to present data about the effects of epoxiconazole on spermatogenesis.

## 2. Materials and Methods

### 2.1. C. elegans Strains and Drug Treatments

Wild-type N2, DR466 (*him-5(e1490)*), and CB4108 (*fog-2(q71)V*), obtained from the *Caenorhabditis* Genetics Center (CGC, University of Minnesota, Minneapolis, MN, USA), were used in the study and maintained at 20 °C as described [23]. Age-synchronized populations of L2-larvae nematodes were obtained by the collection cultured in 20 °C.

Epoxiconazole was purchased from J&K SCIENTIFIC LTD. (Shanghai, China). Epoxiconazole was dissolved in DMSO (Sigma-Aldrich, St. Louis, MO, USA) and M9 buffer (2.5 g of NaCl, 3.0 g of Na_2_HPO_4_ and 1.5 g of KH_2_PO_4_) to prepare the working solution at final concentrations of 0.1, 1.0 and 10.0 μg/L. Epoxiconazole exposures were performed for 48 h in 24-well culture plates at 20 °C according to a previous description [24]. The solvent controls were prepared in the same way.

### 2.2. Outcross Progeny Assay

The number of outcross progeny was counted as previous described [25]. *him-5* mutants were exposed to epoxiconazole for 48 h, then crossed with a young adult *fog-2* female for 12 h. Nematodes were transferred daily to new agar plates until the females ceased egg-laying. Hatched progeny were allowed to grow to L4 stage. The progeny number of F1 generation of this crossing was counted manually. Twenty nematodes were examined in the control and exposed groups. Three replicates were performed.

### 2.3. Germline Staining Assay

Germline counts were performed as described [26]. The male germline was stained with DAPI (4,6-diamidino-2-phenylindole) nucleotide stain followed the procedures as previous described [27]. DAPI-labeled germline were mounted on a glass slide so that sperm nuclei could be viewed under epifluorescence. The germ cell number was then counted by identifying DAPI-stained spermatid nuclei. A mitotic germ cell proliferation assay was performed as described in [28]. The cells within the mitotic region were determined by counting from the row adjacent to the DTC (Distal Tip Cell) to the row containing two or more crescent-shaped nuclei of germ cells, which means the early meiotic prophase I. Ten worms were picked out at indicated time points.

### 2.4. Meiotic Entry Assay

The meiotic entry was observed followed the procedures as previous described [29,30]. The age-matched L2-larvae nematodes were exposed to epoxiconazole for 12 h. Entry into meiosis was confirmed by looking at the first appearance of crescent-shaped nuclei in L3-larvae (12 h after L2) in the mitotic region/transition zone [31]. Ten nematodes were used to calculate the percentage of meiotic entry (meiotic entry worms/total worms × 100).

### 2.5. Sperm Size and Morphology Assay

The sperm size was measured and analyzed as previously published [25]. After exposure to epoxiconazole for 48 h, *him-5* was placed in a drop of SM (Sperm Medium) solution (50 mM of Hepes, 1 mM of MgSO_4_, 25 mM of KCl, 45 mM of NaCl and 5 mM of CaCl_2_ at pH 7.0) and then dissected to release spermatids. A total of 100 spermatids from different fields for each sample were observed and measured under a differential interference contrast (DIC) microscope (Olympus BX41, Tokyo, Japan) [32]. The diameter and cross-sectional area of spermatids were analyzed using Image-Pro Plus 6.0 (Media Cybernetics, Rockville, MD, USA). Ten nematodes were used, and three replicates were performed.

### 2.6. Sperm Activation Assay

The sperm activation was measured according to a previous description [33]. Male *him-5* mutants were exposed to epoxiconazole for 48 h and then dissected to release sperm into SM buffer containing 20 μL of Pronase E (200 μg/mL) on a glass slide under a DIC microscope (Zeiss AX10, Carl Zeiss AG, Oberkochen, Germany). Pronase triggers the process of sperm activation by its proteolytic activity. After 5 min of treatment, sperm activation was observed under a DIC microscope. The activated sperms were scored, and the percentage of activated sperm was calculated as follows: activated sperm/total sperm ×100. Ten nematodes were used, and three replicates were performed.

### 2.7. Sperm Migration Assay

The sperm migration was performed based on a previous description [34]. Observation of mitotracker-labeled male-derived sperm movement within female reproductive tracts was used to analyze the mitotracker sperm migration. Synchronized *him-5* males were labeled and incubated in both MitoTracker Red CMXRos (Invitrogen, Carlsbad, CA, USA) and epoxiconazole solutions for 48 h at 20 °C, then crossed at the ratio of 3:1 with young adult of *fog-2* female for 8 h. Then, the female was transferred to a new plate ensuring enough time for sperms to migrate to the spermatheca before observation. Mitotracker fluorescence was observed by fluorescence microscopy (Olympus FSX100, Olympus, Tokyo, Japan). Only successful mating and corpse integrity worms were practical. The abnormal sperm migration was observed in ectopic positions (uterus and vulva). The percentage of abnormal sperm migration (abnormal migration worms/total worms × 100) was calculated. Ten nematodes were used, and three replicates were performed.

### 2.8. Real-Time Quantitative PCR for Relative Genes Expression Levels

Approximately 6000 male *him-5 in L2-larvae* in each group were exposed to epoxiconazole for 48 h at 20 °C. Trizol (Sigma-Aldrich, St. Louis, MO, USA) was used to extract the total RNA of whole nematodes. A NanoDrop 1000 Spectrophotometer (Thermo scientific, Waltham, MA, USA) was utilized to measure the quantity and quality of RNA, and 260 out of 280 ratios of the samples in our paper are between 1.9 and 2.0. Then, total RNA was converted to synthesize cDNA. Briefly, 1 μg of RNA and 1 μL of 10 mM Oligo dT18 were added to each 1.5 mL tube. A portion of 15 μL of RNase-free water was added and chilled on ice after annealing for 5 min via incubation at 70 °C. The final performed volume of RNA reverse transcription was 25 μL containing 5 μL of the moloney murine leuke virus (MMLV) reaction buffer, 1.25 μL of 4× dNTPs, 0.65 μL of RNase inhibitor, and 1 μL of MMLV (Promega, Madison, WI, USA). The cDNA was synthesized for 60 min at 45 °C and heated for 5 min to 95 °C [25].

The reverse transcription products were measured using SYBR Green I dye (Toyobo, Osaka, Japan). Real-time qRT-PCR was performed in a final volume of 20 μL containing 1 μL of cDNA, 8 μL of SYBR Green I Master Mix (Toyobo, Osaka, Japan), 2 μL of SYBR Green I Master Plus (Toyobo, Osaka, Japan), and 10 mM of each pair of oligonucleotide primers (Invitrogen, Carlsbad, CA, USA) 1.2 μL. The cycle conditions were 94 °C for 5 min, followed by 40 cycles for 5 s at 94 °C and 72 °C for 10 min. The primers were performed and are presented in Table 1. Relative expression levels were determined with Mastercycler gradient PCR (Eppendorf, Hamburg, Germany) and ABI StepOne Quantitative PCR (ABI, Carlsbad, CA, USA).

Comparative Ct was used to compare mRNA levels with separate tubes. *act-1*, consistently expressed at all stages in *C. elegans* [23] and which exhibited relatively stable expression as a reference gene when exposed to chlorpyrifos in our previous paper [25], was used as an internal control gene to normalize individual samples [35]. The presence of primer dimers and nonspecific PCR products were evaluated via dissociation curve analysis. The expression stability of *act-1* was confirmed by no differences in Ct value among the groups after a constant amount of RNA was added to each reverse transcription reaction. For the ΔΔCt calculation to be valid, the amplification efficiencies of the target and reference were found to be approximately equal in our paper. Each analysis was conducted by three replicates.

### 2.9. Statistical Analysis

Data were plotted as means ± standard error of the mean (SEM). One-way ANOVA and Dunnett’s *t*-test were used in the study for comparison between the control and the exposed groups, while a non-parametric was performed to analyze data if unequal variance was found. Statistical analysis was processed using SPSS 13.0 (SPSS Inc., Chicago, IL, USA). Probability levels of 0.05 were considered statistically significant.

## 3. Results

### 3.1. Assessment of Reproductive Capacity in Epoxiconazole-Exposed Male him-5

**Figure 1:**
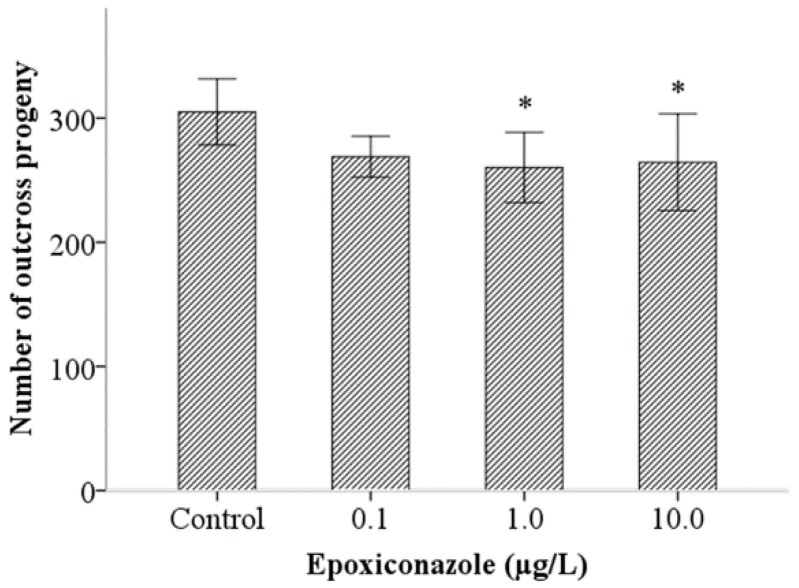
Effects of epoxiconazole exposure on the number of outcross progeny. Bars represent means ± SEM (standard error of the mean). * *p* < 0.05 vs. the control group.

First, the number of outcross progeny in the *him-5* mutant was counted to analyze male fertility. As shown in Figure 1, despite substantial overlap in the variance between groups, the number of outcross progeny in *him-5* significantly decreased in the groups treated with 1.0 μg/L and 10.0 μg/L epoxiconazole (*p* < 0.05). It showed that epoxiconazole exposure in *C. elegans* can reduce male outcross progeny, indicating that epoxiconazole may cause potential germ cell damage and disturb spermatogenesis.

**Figure 2:**
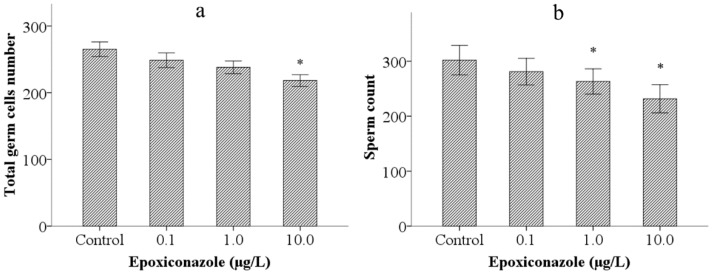
Effects of epoxiconazole exposure on total germ cells and spermatids. (**a**) Number of total germ cells nucleus; (**b**) Number of spermatids nucleus. Bars represent means ± SEM. * *p* < 0.05 vs. the control group.

Second, to analyze the possible reasons inducing the reduction of male outcross progeny in *him-5*, we investigated effects of epoxiconazole exposure on total germ cells. The results showed that exposure to 10.0 μg/L epoxiconazole significantly decreased the number of total germ cells (*p* < 0.05) (Figure 2a).

Third, we observed sperm count (the number of spermatids) in epoxiconazole exposed male *him-5* mutants. As shown in Figure 2b, the number of spermatids in male *him-5* from the groups treated with 1.0 μg/L and 10.0 μg/L epoxiconazole significantly decreased compared with those of the control (*p* < 0.05).

### 3.2. Effects of Epoxiconazole Exposure on Mitotic Cells

**Figure 3:**
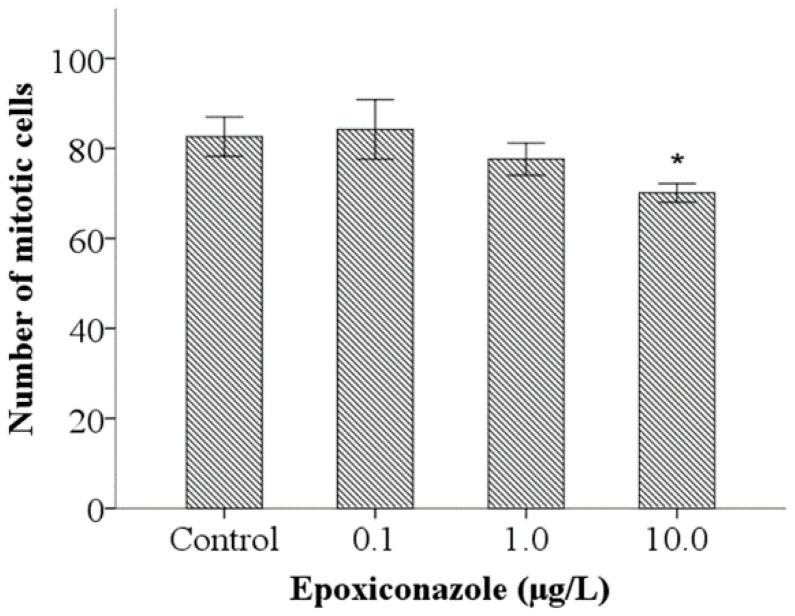
Effects of epoxiconazole exposure on mitotic cells. Bars represent means ± SEM. * *p* < 0.05 vs. the control group.

As shown in Figure 3, compared with that of the control, the number of mitotic cells in 10.0 μg/L epoxiconazole-exposed male *him-5* mutants was significantly decreased (*p* < 0.05), which indicated that germ cell proliferation in nematodes was inhibited by epoxiconazole exposure.

### 3.3. Effects of Epoxiconazole Exposure on Meiotic Cells

**Figure 4:**
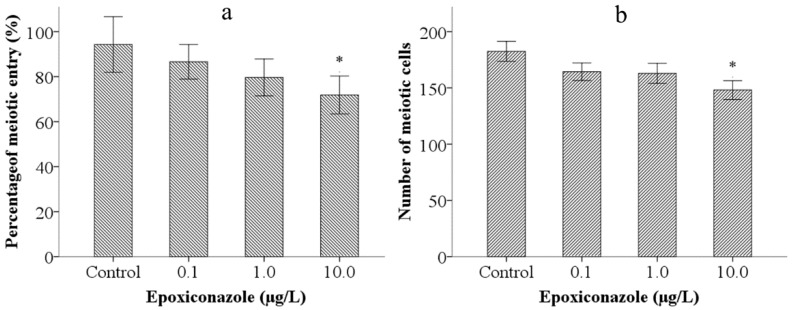
Effects of epoxiconazole exposure on meiotic cells. (**a**) Percentage of meiotic entry; (**b**) Number of meiotic cells. Bars represent means ± SEM. * *p* < 0.05 vs. the control group.

Meiotic entry test of male nematodes was employed to analyze effects on germ cell differentiation induced by epoxiconazole. As shown in Figure 4a, compared with that of the control, the percentage of meiotic entry in nematodes was significantly declined (*p* < 0.05) after exposure to epoxiconazole at a concentration of 10.0 μg/L. Further, the number of meiotic cells in male *him-5* from the groups treated with 10.0 μg/L of epoxiconazole significantly decreased compared with that of the control (*p* < 0.05) (Figure 4b). The results indicated that epoxiconazole induces premature entry into meiosis and reduces the number of meiotic cells.

### 3.4. Effects of Epoxiconazole Exposure on Sperm Size

**Figure 5:**
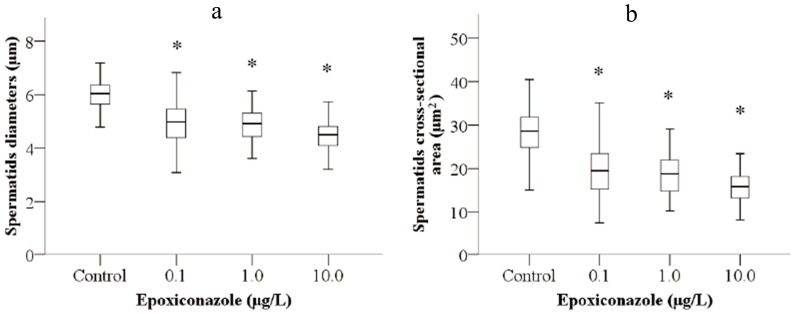
Effects of epoxiconazole exposure on sperm size. (**a**) Spermatid diameter; (**b**) Spermatid cross-sectional area. Bars represent means ± SEM. * *p* < 0.05 vs. the control group.

The diameter and cross-sectional area of spermatids were used to evaluate the size of sperm. As shown in Figure 5a, exposure to 0.1–10.0 μg/L epoxiconazole all significantly decreased spermatids diameter of male *him-5* mutants compared with that without epoxiconazole exposure (*p* < 0.05). Similarly, the significant decreases of cross-sectional area were observed in male *him-5* nematodes exposed to 0.1–10.0 μg/L epoxiconazole (*p* < 0.05) (Figure 5b).

### 3.5. Effects of Epoxiconazole Exposure on Sperm Activation and Sperm Migration

**Figure 6:**
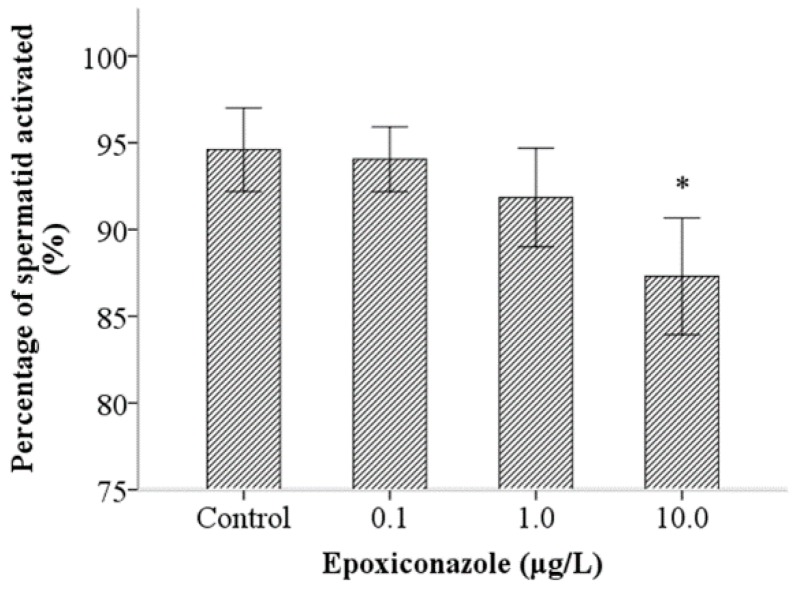
Effects of epoxiconazole exposure on sperm activation. Bars represent means ± SEM. * *p* < 0.05 vs. the control group.

**Figure 7:**
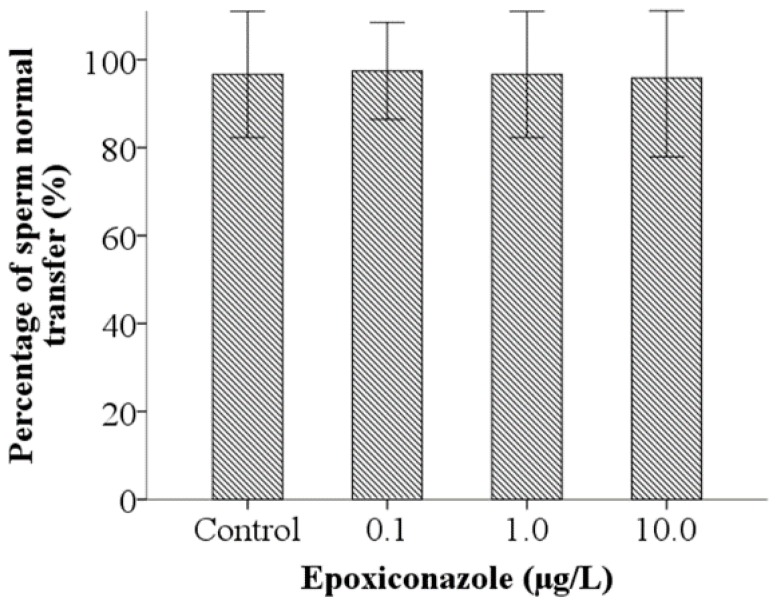
Effects of epoxiconazole exposure on sperm migration.

A sperm activation assay was used to investigate the ability of sperm motility. As shown in Figure 6, the percentage of spermatid normal activation, compared with that of the control, were significantly decreased when exposed to epoxiconazole at a concentration of 10.0 μg/L (*p* < 0.05), which indicates that sperm activation was inhibited by epoxiconazole exposure. However, the results of the percentage of sperm normal transfer were not evidently altered between exposed nematodes and control nematodes (*p* > 0.05) (Figure 7).

### 3.6. Effects of Epoxiconazole Exposure on TGF-β Pathway Gene Expression

**Figure 8:**
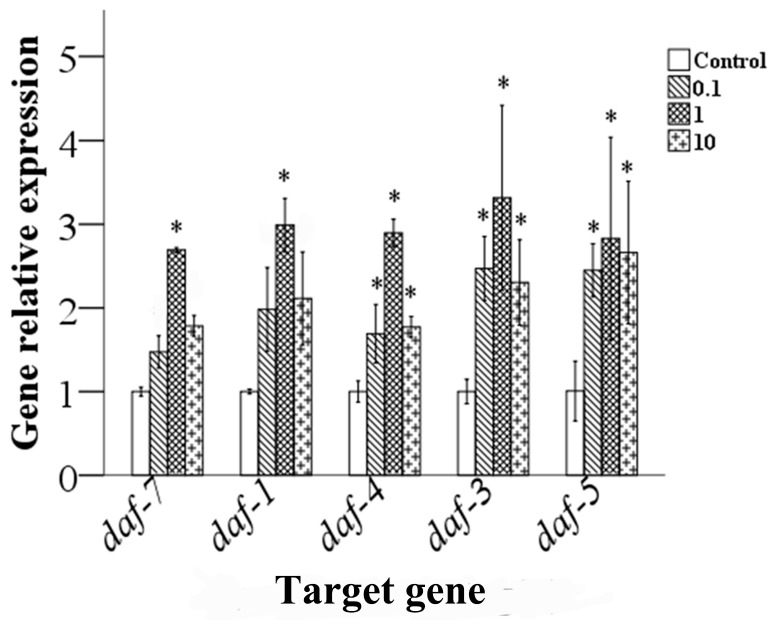
Effects of epoxiconazole exposure on the expression levels of TGF-β signaling pathway genes. Bars represent means ± SEM. * *p* < 0.05 vs. the control group.

The TGF-β signaling pathway is involved in germ cell development and spermatogenesis in *C. elegans*. Therefore, we analyzed expression levels of a gene associated with the TGF-β signaling pathway. As shown in Figure 8, the results indicate that, compared with that of the control, the expression levels of gene *daf-3*, *daf-4* and *daf-5* in nematodes exposed to 0.1–10.0 μg/L epoxiconazole were significantly increased (*p* < 0.05), while expression levels of gene *daf-*1 and *daf-7* were significantly increased at a concentration of 1.0 μg/L (*p* < 0.05).

## 4. Discussion

Spermatogenesis of the nematode is highly similar to that of mammals, undergoing mitosis, meiosis to produce motility sperm [20,26]. Therefore, the effect of chemicals on spermatogenesis in *C. elegans* might reflect the damage in mammals to a certain extent [20]. In this study, we investigated potential damage in the germline and found that male outcross progeny, the total number of germ cells, and spermatids can be reduced following epoxiconazole exposure, a finding that is similar to that reported by Grote et al. [9]. Based on this, we assume that epoxiconazole can reduce the number of spermatids and germ cells and result in abnormalities of germ cell development. There is no physiological germ cell apoptosis to maintain the male germline during germ cell development [36,37]. Therefore we focused on the number of meiotic cells in the adult male nematode and the meiosis entering time to illustrate germ cell differentiation. The results show that epoxiconazole induced abnormalities in the process of germ cell differentiation and reduced the number of meiotic germ cells. Possible reasons for this may be that there are a large number of genes associated with cytochrome P450 (CYP450) in *C. elegans*. Epoxiconazole can affect the activity of CYP450 and inhibit steroidogenic processes such as meiosis-activating sterols to disrupt meiosis [12,38]. In addition, epoxiconazole acts as an endocrine-disrupting chemical (EDC), affecting the production of sex hormones that play an important role in spermatogenesis. A study by Hoss shows that many processes are regulated via hormonal pathways in nematodes [39]. Therefore, we are interested in whether epoxiconazole may affect the activity of CYP450 and induce disturbances in spermatogenesis in *C. elegans*; however, the specific sites of action still need further study.

After completion of the meiotic divisions, spermatids develop into motile spermatozoa, a process referred to as spermiogenesis [40]. Our results showed that epoxiconazole exposure can lead to noticeable alterations in the size of sperm, suggesting that epoxiconazole may have effects on the process of spermatid budding. Sperm size is associated with sperm competition, and larger sperm outcompete smaller sperm prior to fertilization in the nematode *C. elegans* [41], suggesting that epoxiconazole may cause a decline in sperm competitiveness and consequently in male fertility. Next, we investigated the effects of sperm activation associated with sperm motility. The results show that epoxiconazole exposure can inhibit sperm activation, suggesting that epoxiconazole may have effects on the morphogenesis of sperm pseudopodia. Sperm activation is affected by various factors, one of which is cholesterol. A previous study indicated that triazole fungicides act as inhibitors of certain pathways of steroidogenesis and can affect the content of cholesterol [42]. In addition, the iron channel is also a kind of factor affecting sperm activation. It was reported that triazole fungicides can non-specifically inhibit calcium channels and disturb intracellular Ca^2+^ [43,44]. All of these may be mechanisms by which epoxiconazole causes abnormal sperm activation.

Further, we explored the effects of epoxiconazole on sperm migration, but no evident alterations were observed in this study. Sperm migration is affected by various factors, one of which is the generation of sperm pseudopodia, because pseudopod morphology and functions are directly related to sperm motility. The sperm activation assay showed that epoxiconazole induced an increase in abnormal sperm pseudopodia, suggesting that sperm motility may be damaged. Sperm size is also one of the factors that can affect sperm motility, since larger sperm swim faster and displace smaller sperm, taking precedence at fertilization [41].

At present, the mechanism underlying the reproductive toxicity of epoxiconazole is mostly focused on endocrine interference, but the specific regulatory mechanism of spermatogenesis is still less involved. Therefore, our study focused on the TGF-β signaling pathway associated with germ cell development in *C. elegans* after epoxiconazole exposure. Several authors have reported that the TGFβ receptor responds in the DTC by modulating transcriptional targets that influence the proliferation/differentiation decision (mitosis versus meiosis) in the germ line either directly or indirectly in *C. elegans* [45,46]. It was reported that the TGFβ/DAF-7 pathway promotes sperm targeting of spermathecae and modulates sperm motility critical for fertilization; down-regulating TGFβ signaling in young adults causes sperm-targeting defects [47]. The analysis of data in our study shows that epoxiconazole activates the TGFβ signaling pathway and increases gene expression levels. Combined with the inhibition of germ cell proliferation, this suggests that the TGF-β signaling pathway may be one of the ways in which spermatogenesis is impaired by epoxiconazole.

In addition, we found that the expression of TGF-β signaling pathway genes was different when treated with concentrations of 1.0 or 10.0 μg/L of epoxiconazole. The relative gene expression levels were higher at a concentration of 1.0 μg/L than in other groups, while the number of germ cells was reduced at a concentration of 10.0 μg/L. The reason may be that the mRNA reaction is relatively more sensitive, and results in differential expression at low concentration. However, there may be some delayed phenomenon acting at the cell level especially in the physiological process in nematodes through a series of processes such as mRNA transcription, protein translation, and related signaling pathway activation. Thus, the toxic effects of epoxiconazole are observed at a concentration of 10.0 μg/L.

## 5. Conclusions

In summary, we demonstrated that epoxiconazole activates the TGFβ signaling pathway, induces mitotic germ cell proliferation arrest and premature entry into meiosis, reduces the total number of germ cells, mitotic cells, meiotic cells and spermatids, as well as spermatid diameter and cross-sectional area, and inhibits sperm activation in male *C. elegans*, eventually leading to impaired spermatogenesis and a decline in fertilization. Although the study identified a potential signaling pathway involved in modulating abnormalities of epoxiconazole-induced spermatogenesis, more related mechanisms should be further explored.

## Figures and Tables

**Table 1:** Gene primers tested in the study.

Gene Name	Forward Primer	Reverse Primer
*act-1*	ATGTGTGACGACGAGGTT	GAAGCACTTGCGGTGAAC
*daf-7*	TTACGAGAAGAACGAGGATG	TTGGAAGTTGAATGCTGATAC
*daf-1*	GTTGCTGGACAAGAAGGC	ACCAAGAAGTGGGCGTGA
*daf-4*	GGTGATGAGTATTGGATTGTG	ATTGGCTTCTTTGGGTGT
*daf-3*	TTACAACCATCAACAGTCACC	TCCAAAACCTCACCGTCT
*daf-5*	CGAAAACCTCAACATCACA	CATCCTCCTCCAAGTCATC
